# Mixed Methods Survey of Zoonotic Disease Awareness and Practice among Animal and Human Healthcare Providers in Moshi, Tanzania

**DOI:** 10.1371/journal.pntd.0004476

**Published:** 2016-03-04

**Authors:** Helen L. Zhang, Kunda W. Mnzava, Sarah T. Mitchell, Matayo L. Melubo, Tito J. Kibona, Sarah Cleaveland, Rudovick R. Kazwala, John A. Crump, Joanne P. Sharp, Jo E. B. Halliday

**Affiliations:** 1 Duke University Medical Center, Durham, North Carolina, United States of America; 2 Nelson Mandela African Institute of Science and Technology, Arusha, Tanzania; 3 Sokoine University of Agriculture, Morogoro, Tanzania; 4 Centre for International Health, University of Otago, Dunedin, New Zealand; 5 Institute of Biodiversity, Animal Health and Comparative Medicine, University of Glasgow, Glasgow, United Kingdom; 6 Kilimanjaro Christian Medical Centre, Moshi, Tanzania; 7 Kilimanjaro Christian Medical University College, Moshi, Tanzania; 8 School of Geographical and Earth Sciences, University of Glasgow, Glasgow, United Kingdom; Institute of Tropical Medicine, BELGIUM

## Abstract

**Background:**

Zoonoses are common causes of human and livestock illness in Tanzania. Previous studies have shown that brucellosis, leptospirosis, and Q fever account for a large proportion of human febrile illness in northern Tanzania, yet they are infrequently diagnosed. We conducted this study to assess awareness and knowledge regarding selected zoonoses among healthcare providers in Moshi, Tanzania; to determine what diagnostic and treatment protocols are utilized; and obtain insights into contextual factors contributing to the apparent under-diagnosis of zoonoses.

**Methodology/Results:**

We conducted a questionnaire about zoonoses knowledge, case reporting, and testing with 52 human health practitioners and 10 livestock health providers. Immediately following questionnaire administration, we conducted semi-structured interviews with 60 of these respondents, using the findings of a previous fever etiology study to prompt conversation. Sixty respondents (97%) had heard of brucellosis, 26 (42%) leptospirosis, and 20 (32%) Q fever. Animal sector respondents reported seeing cases of animal brucellosis (4), rabies (4), and anthrax (3) in the previous 12 months. Human sector respondents reported cases of human brucellosis (15, 29%), rabies (9, 18%) and anthrax (6, 12%). None reported leptospirosis or Q fever cases. Nineteen respondents were aware of a local diagnostic test for human brucellosis. Reports of tests for human leptospirosis or Q fever, or for any of the study pathogens in animals, were rare. Many respondents expressed awareness of malaria over-diagnosis and zoonoses under-diagnosis, and many identified low knowledge and testing capacity as reasons for zoonoses under-diagnosis.

**Conclusions:**

This study revealed differences in knowledge of different zoonoses and low case report frequencies of brucellosis, leptospirosis, and Q fever. There was a lack of known diagnostic services for leptospirosis and Q fever. These findings emphasize a need for improved diagnostic capacity alongside healthcare provider education and improved clinical guidelines for syndrome-based disease management to provoke diagnostic consideration of locally relevant zoonoses in the absence of laboratory confirmation.

## Author Summary

Zoonoses are diseases that are naturally transmitted between animals and humans. In Tanzania, research has shown that several zoonoses, including brucellosis, leptospirosis, and Q fever, are common, but under-diagnosed, causes of human illness. We conducted a mixed methods survey, combining quantitative and qualitative research techniques, of healthcare providers in Moshi, Tanzania. Four (40%) of 10 animal sector respondents and 15 (29%) of 52 human sector respondents reported seeing brucellosis cases in the past 12 months, while none reported cases of leptospirosis or Q fever. Nineteen (31%) respondents reported awareness of a local diagnostic test for human brucellosis, while one reported locally available diagnostic tests for human leptospirosis and Q fever. One (2%) respondent reported a locally available animal brucellosis test, and none reported tests for leptospirosis or Q fever in animals. Many respondents mentioned a lack of diagnostic testing resources during interviews. Our findings suggest that diagnostic testing capacity improvements alongside public health campaigns and healthcare provider education are key steps toward improving recognition and accurate diagnosis of zoonoses in this setting. An improved understanding of healthcare provider awareness, perceptions, and practices regarding zoonoses is critical for the design of effective programs to improve diagnosis and treatment of these diseases.

## Introduction

Zoonoses are important causes of human and animal morbidity [1,2]. Of the known pathogen species implicated in human disease, 61% are zoonotic [3]. Comparatively neglected among this vast group of pathogens are endemic zoonoses, which, despite their low profile within the global health agenda, have tremendous health and economic implications in the developing world. Many endemic zoonoses not only cause considerable human disability but also impair livestock productivity, imposing multiple burdens on poor communities [1,4].

Northern Tanzania is representative of many settings with close human-livestock interactions [5], where endemic zoonotic diseases are common yet under-recognized etiologies of human illness. In a prospective cohort study of 870 inpatients conducted in 2007–2008 in the Kilimanjaro Region of Tanzania, bacterial zoonoses accounted for more than a quarter of febrile hospital admissions. Specific zoonoses identified included brucellosis in 3.5% of febrile inpatients, leptospirosis in 8.8%, and Q fever in 5.0% [6–9]. Despite the high prevalence of bacterial zoonoses observed using study diagnostics, none of the study patients received a clinical diagnosis of a bacterial zoonosis. Instead, malaria was clinically diagnosed in 60.7% of this cohort despite being the actual cause of fever in only 1.6% [9]. The number of ‘missed’ cases of zoonotic disease supports the view that the compound impacts of many zoonoses are likely to be under-documented by existing surveillance systems, resulting in gross underestimates of local, regional, and ultimately global disease burdens [2,10,11].

The recognition and successful clinical management of zoonoses depend on several factors, including individual healthcare seeking behavior; differential consideration of a zoonosis by patients and clinicians; availability, accuracy, and uptake of diagnostic testing; and treatment accessibility. Clinical recognition of many zoonoses is made difficult by their nonspecific clinical presentations that overlap with other febrile illnesses. This may partly explain the frequent misattribution of febrile zoonoses to pathogens for which there is greater awareness, such as malaria and typhoid [9]. It is widely recognized that limited testing capacity (considering both test availability and performance) in sub-Saharan Africa severely limits the options for accurate laboratory diagnostic testing for zoonoses [12].

Previous studies in northern Tanzania have indicated that clinicians’ perceptions of patient pressure play a role in malaria over-diagnosis and over-treatment, suggesting a need to raise awareness about alternative causes of febrile illness, including zoonoses, among both the healthcare provider and patient populations [13,14]. The few studies conducted to date reveal limited awareness of zoonoses among health workers [15–17]. However, little is known about the degree to which zoonotic diseases are considered or recognized by healthcare providers, or about factors influencing the diagnostic consideration of zoonoses in human or animal populations. Understanding this relationship is an essential step toward improving the quality of care for patients presenting with febrile illness as well as achieving adequate livestock disease control.

A small number of studies have successfully applied mixed methods designs to investigate the intersection of human and animal health [18–20]. When adequately employed, this approach has the advantage of addressing the gaps in understanding that are left un-broached in strictly quantitative approaches.

We used a mixed methods approach for this study, employing an embedded study design [21,22]. This involved performing a largely quantitative questionnaire survey to examine medical and veterinary healthcare providers’ perceptions, practices, knowledge, and recent experiences of several named zoonotic diseases in Moshi, northern Tanzania. Immediately following each survey, semi-structured interviews were conducted with the questionnaire respondents. These involved discussion of the findings of a previous fever etiology study by Crump et al. [9] and were used to gather qualitative data to explore health care providers’ explanations for the quantitative survey findings of this study, using the findings of the previous study to prompt discussion. The specific aims of this study were to assess awareness and knowledge among healthcare providers regarding selected zoonoses that have been shown to be common in northern Tanzania, to determine what diagnostic and treatment protocols were available and utilized locally, and to obtain insights into the contextual factors that contribute to the apparent under-diagnosis of zoonoses in this area.

## Methods

### Study area

Moshi is located in the Kilimanjaro Region of northern Tanzania. Moshi municipality is subdivided into 21 wards and has a total population of approximately 184,000 people [23]. It is considered to have low malaria transmission intensity [7]. Dominant livestock populations in the Kilimanjaro Region comprise cattle, goats, sheep, and pigs [5]. Within the predominantly urban wards of Moshi, livestock production is generally practiced in smallholder units [24].

### Healthcare sector mapping and respondent selection

We conducted an initial survey of the formal animal and human healthcare provider communities serving Moshi (e.g. within the 21 wards of Moshi municipality) to describe the structure of these sectors and develop a sampling frame. In the animal healthcare sector, government-appointed Livestock Field Officers (LFO) operate at ward level and serve as the frontline public-sector providers of veterinary services, meat inspections, community education on livestock care, as well as data compilation from livestock keepers on livestock diseases and vaccinations for official reporting. In the human healthcare sector, formal clinical services are provided through registered dispensaries, health centers, and hospitals. Additionally, government-appointed Ward Environmental Health Officers (WEHO) provide human disease prevention services, community health education, and case reporting of human diseases by healthcare facilities to the municipal government. The target populations for our questionnaire and interview study included all government registered individuals and facilities providing primary livestock veterinary care and advice to livestock keepers, and primary care and advice to individuals with febrile illness.

We attempted to contact all identified LFOs and WEHOs directly. At healthcare facilities, candidate study participants were defined as registered healthcare providers (e.g. medical doctors, medical officers, assistant medical officers, clinical officers, and nurses) who provided care and advice to individuals with undifferentiated febrile illness. Specialty registered healthcare facilities, such as maternity units and dentists, were excluded. At facilities with a single candidate participant (e.g. all dispensaries and most health centers), that individual was approached for study participation. At facilities with 2–5 candidate participants, the individual with the greatest direct contact with care-seekers was approached. At hospitals, study team members first met with a senior physician or administrator to obtain a list of all candidate healthcare providers working in departments admitting patients presenting with fever. All listed individuals who were available to participate on pre-scheduled dates were approached. Written informed consent was sought from all respondents prior to study participation. One ward was selected as a pilot for trial of survey procedures; data collected in this ward were excluded from analysis.

### Questionnaire administration

The study questionnaire was administered orally in Swahili. Questionnaires included close-ended and free-response questions covering the following topics: respondent training; general zoonoses knowledge; reported recent experience of zoonoses cases; knowledge of signs and symptoms of selected zoonoses in humans and animals; knowledge of zoonoses transmission; and testing, prevention, and treatment practices (S1 Questionnaire). The section assessing knowledge of disease symptoms and signs consisted of a series of closed (yes/no response) questions asking whether a particular symptom or sign was commonly associated with each disease. Listed symptoms and signs included those commonly associated with each named disease as well as others not typically associated with any of these diseases.

### Semi-structured interviews

All respondents were invited to participate in an open-ended interview and provide feedback immediately following questionnaire completion (S1 Interview Schedule). In each interview, the study team member described the rationale for the current study and provided summary results of a study conducted in Moshi in 2007–8 to determine etiologies of febrile illness in this area (see [9] for further details of this study). The key study findings that were communicated to the respondent were: the low prevalence of confirmed malaria among hospitalized febrile patients (2%); the high proportion of febrile patients that were clinically diagnosed with malaria (61%); the higher confirmed prevalence of several zoonoses including brucellosis (4%), leptospirosis (9%), and Q fever (5%); and finally, the fact that none of the study patients were clinically diagnosed with a bacterial zoonosis. Respondents were then asked a series of questions about these findings, including: whether the proportions of patients diagnosed with malaria and zoonotic diseases were higher or lower than they would have thought; why they thought that so few patients were initially diagnosed with any zoonoses; and if they had any comments or questions about the study findings presented. The participant’s reactions to these data and their responses to the questions asked were either audio recorded, when possible, or key points were written down. Study team members then transcribed the audio recordings and translated all transcriptions and handwritten notes from Swahili to English for content analysis.

### Analysis of questionnaire data

Questionnaire responses were recorded and entered into a database using TeleForm 9.0 (Cardiff Inc., Vista, CA, USA). Data were analyzed using R v3.1.1 in RStudio v0.98.1074 (Boston, MA, USA) [25]. Fisher’s exact test was used for pairwise comparisons. Cohen’s kappa was used to assess agreement between the disease symptoms and signs reported by respondents and those included in a CDC case definition used as a reference expert opinion [26]. Generalized linear models with binomial errors were used to examine the relationship between participant responses about clinical signs of brucellosis (the proportion of responses in agreement with the expert opinion) and potential explanatory variables of interest.

### Analysis of interview data

Thematic analysis approaches were used to identify, analyse and report themes within the qualitative dataset collected [27]. Two members of the study team (STM and KWM) read through all interview records to become familiar with their content, and JEBH and JPS also read through a sample of records. Themes and codes were then developed to capture the salient features of the data. Codes were initially developed independently by STM, KWM, JEBH, and JPS. This team included members with medical, epidemiological, human geography, and public health research training. The code development process emerged both from the conceptual knowledge of the research team and from the analysis of interview material [28]. In this case, it reflected pre-conceived approaches to the classification and synthesis of data e.g. codes for the comparison of respondent experience as compared to the findings of the previous etiology study and codes specific to different steps in the process of zoonoses recognition and reporting. Code development also reflected the team members’ awareness of the existing literature on malaria over-diagnosis. In addition, more general codes were included to capture less clearly pre-defined content about the roles of individual knowledge and the broader environment in the clinician-patient interaction. Code development was focused to extract data relevant to the respondents’ understanding of the causation of the quantitative data recorded and discussed. Codes developed by the four researchers were then compared and a final consensus code set agreed through discussion between study team members (Table 1). STM and KWM then re-read the written records and independently applied these codes to each, recording in each case the support or not of each code by the interview content. In cases where these reviewers disagreed, JPS acted as tiebreaker to achieve the final dataset [28]. Overall summaries of the datasets were made with reference to the sector (animal or human health) of the respondents.

**Table 1:** Themes and codes identified as factors influencing the recognition and reporting of zoonoses and applied to synthesize the records of feedback interviews with each respondent (n = 59).

		Respondent Sector			
		Livestock		Human health	
		n	(%)	n	(%)
**RESPONSE TO RESEARCH FINDINGS**					
Malaria figures from study were lower than expected		4	(50)	16	(31)
Malaria figures from study were as expected		4	(50)	32	(63)
Zoonoses figures from study were higher than expected		2	(25)	18	(35)
Zoonoses figures from study were as expected		4	(50)	16	(31)
**CODE**	**SUB-CODE**				
Knowledge and education	Healthcare providers think that fever = malaria	4	(50)	13	(25)
	General public think that fever = malaria	2	(25)	5	(10)
	Patient pressure to prescribe anti-malarials	1	(13)	0	0
	Existing patient numbers/records proves malaria numbers	0	(0)	4	(8)
	Research not getting back to the healthcare providers	0	(0)	5	(8)
	Healthcare providers lack knowledge of zoonoses	7	(88)	37	(73)
	General public lack knowledge of zoonoses	3	(38)	26	(51)
	Practices changed as a result of knowledge of disease prevention	1	(13)	4	(8)
Technology	Lack of trust in mRDT	0	(0)	12	(24)
	Other tests confirm malaria	0	(0)	11	(22)
Environment	Environment of northern Tanzania conducive to malaria	1	(13)	4	(8)
	Environment of northern Tanzania not conducive to malaria	1	(13)	2	(4)
	Environment of northern Tanzania conducive to zoonoses	1	(13)	10	(20)
	Visibility of vectors	0	(0)	5	(10)
	Movement of animals in region	0	(0)	0	(0)
Morality	Dirt / lack of care as cause of disease	0	(0)	1	(2)
	Disease coming to region from elsewhere	0	(0)	8	(15)
	Lack of knowledge of disease elsewhere	0	(0)	1	(2)
Resources	Lack of resources for tests for zoonoses	5	(63)	41	(80)
	Misuse of resources for tests for zoonoses	1	(13)	5	(10)

### Integration of quantitative and qualitative data

The qualitative data collection for this study was embedded within the quantitative data collection process to facilitate understanding of the reasons for respondents holding the beliefs they offered in the first stage of research. Providing respondents with the opportunity to reflect on the findings of the previous study and their own responses in the quantitative survey revealed aspects of their understanding, reasoning and perceptions [28], in addition to the information on their knowledge and awareness collected through the quantitative phase. This recognition of the socially constructed and contextual nature of knowledge recognizes that institutions, practices and beliefs create the world within which people work [29]. This interpretative stage of research is focused on uncovering and interpreting meanings held by the healthcare providers that “make it ‘rational’ to act in a particular way” [30,31]. Thus, the qualitative data were collected to add richness to our understanding of the factors that operate to produce the patterns of healthcare providers’ awareness of zoonoses and the diagnostic and treatments practice described by the quantitative data collected. Content analysis was used to draw codes from participants’ explanations, in addition to the codes introduced from the researchers’ knowledge of the relevant literature. The analyses of the quantitative and qualitative datasets were conducted in parallel but were not formally integrated as the two datasets were gathered to address linked but non-identical questions.

### Ethics statement

Written informed consent for questionnaire administration and interview participation was obtained from all respondents. The protocols and consent forms used for this study were reviewed and approved by the Research Ethics Committee of the Kilimanjaro Christian Medical Centre (#535), the Tanzanian National Institute of Medical Research National Research Ethics Coordinating Committee, and the Institutional Review Board for Clinical Investigations of Duke University Health System in the United States (Pro00037356).

## Results

### Respondent characteristics

The initial survey yielded details for 15 LFOs serving 14 wards, 17 WEHOs serving 17 wards, and 56 healthcare facilities. All listed individuals and facilities were approached for study participation, apart from those from the pilot ward. A total of 62 individuals were enrolled in the study between May 2014 and February 2015. These comprised 10 LFOs, 9 WEHOs, and 43 clinical staff from 34 health facilities. Reasons for non-participation included repeatedly failed contact attempts, out-of-operation or non-existent facilities, lack of healthcare provision to patients with undifferentiated febrile illness, declining participation, and exclusion due to direct involvement in a previous fever etiology study.

The median (range) respondent age was 42 (23–81) years and 23 (37%) were female. Of 62 respondents, 61 (98%) had attended college or university. Ten (16%) worked in the animal healthcare sector; all were LFOs. Of the 52 (84%) respondents working in the formal human health sector, 9 (17%) were WEHOs and the remaining 43 (83%) were clinical staff practicing in health facilities. Thirty-two (74%) human healthcare providers were based in dispensaries or health centers, 6 (14%) at a private hospital, and 5 (12%) at a public hospital.

### Awareness of zoonoses

**Fig 1:**
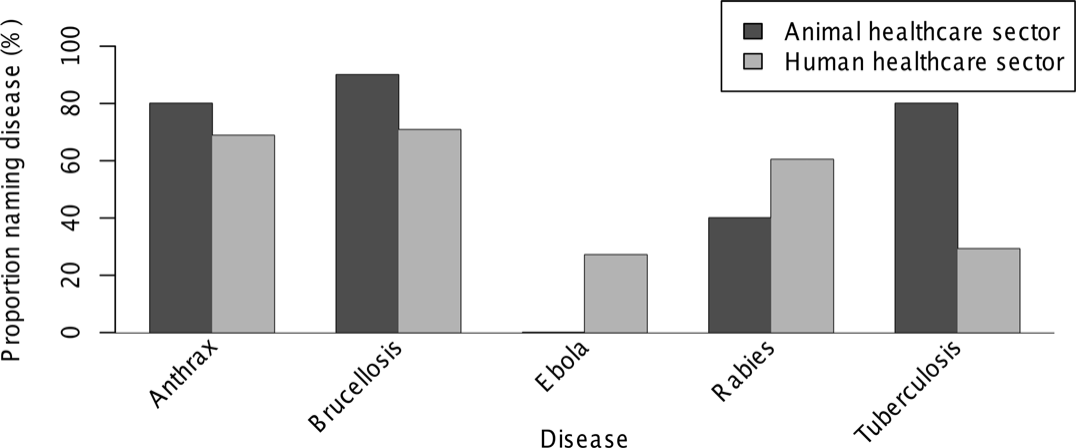
Diseases transmitted from livestock to humans named by animal (n = 10) and human (n = 48) healthcare providers in Moshi, Tanzania, 2014–2015. Named diseases reported by ten or more respondents are included. Bars show proportion of respondents from each sector (out of those responding “Yes” to the question “Do you know of any diseases that people can catch from livestock?”) who reported each disease.

Fifty-eight of 62 (94%) respondents reported knowledge of one or more diseases that could be transmitted from livestock to humans. The most frequently reported diseases and the breakdown of reports by respondent sector are shown in Fig 1. Knowledge of one or more diseases transmitted by rodents was reported by 52 (84%) respondents, of whom 48 (92%) named plague. All (100%) respondents named rabies as a disease that could be transmitted by dogs. In total, 38 (61%) respondents named one or more diseases that could cause abortions in livestock, including 28 (74%) brucellosis, 7 (18%) anthrax, and 4 (11%) Rift Valley fever.

**Fig 2:**
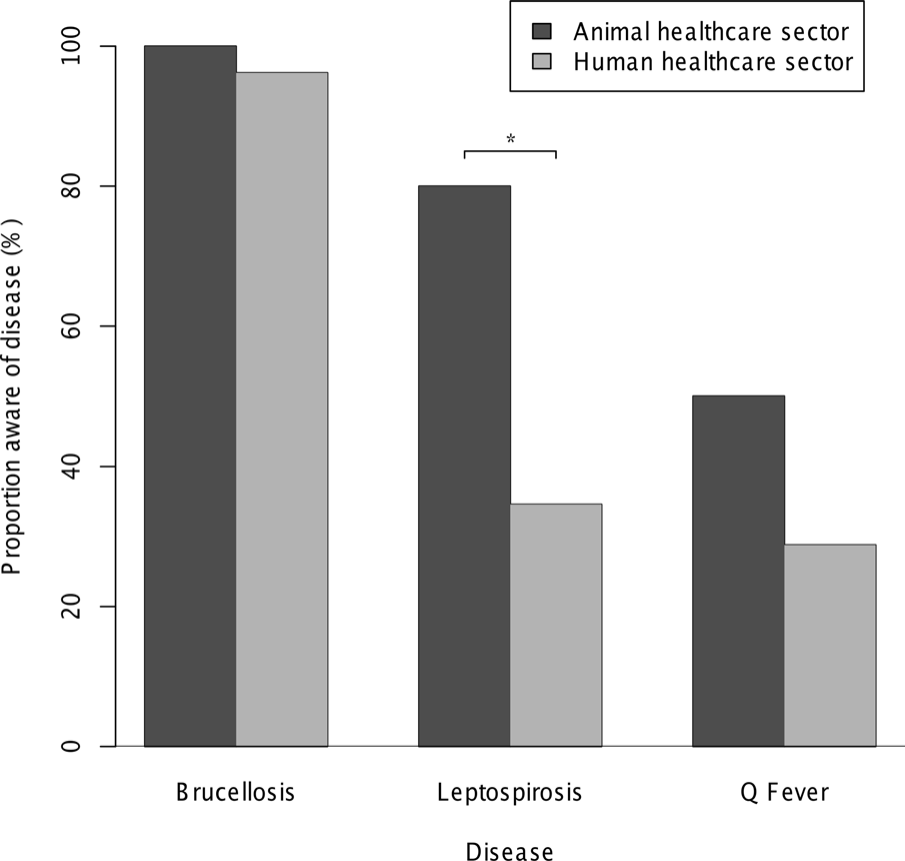
Bacterial zoonoses awareness among healthcare providers in Moshi, Tanzania, 2014–2015. Proportion of animal (n = 10) and human (n = 52) healthcare providers who reported having heard of specific zoonoses (* indicates a significant difference between the proportion of respondents in the two sectors p< 0.05).

Fig 2 depicts awareness by sector of specific named zoonoses. Respondents working in the animal healthcare sector were significantly more likely to have heard of leptospirosis than those in the human healthcare sector (Odds Ratio [OR]: 7.3, 95% Confidence Interval [CI]: 1.3–77.6, p = 0.013). There were no significant differences between sectors in awareness of brucellosis or Q fever (Fig 2).

**Fig 3:**
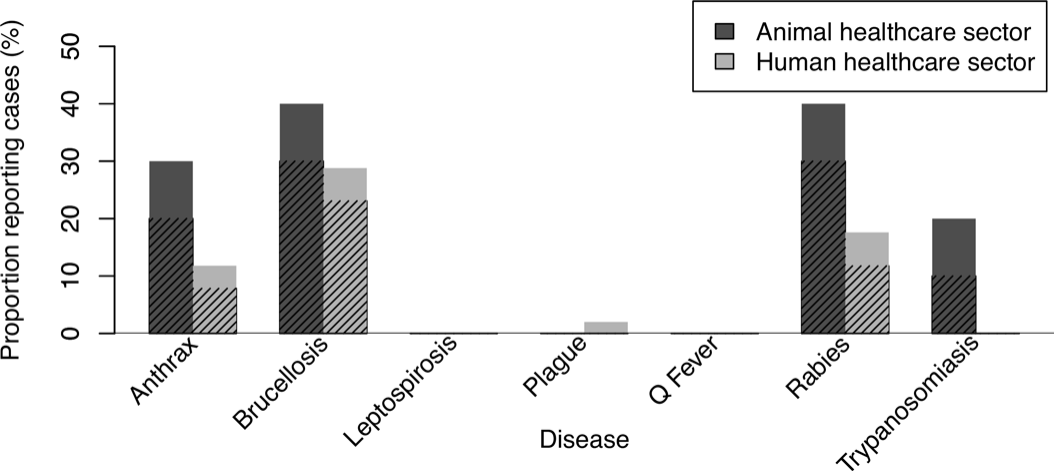
Zoonoses cases reported by healthcare providers in Moshi, Tanzania, 2014–2015. Proportion of animal (n = 10) and human (n = 52) healthcare providers who reported having seen or advised on specific zoonotic diseases during the past 12 months. Respondents were prompted to respond for each named disease. Shading indicates the proportion of all responses that were volunteered before prompting on each specific disease.

Fig 3 shows the proportions of animal and human healthcare providers who reported seeing or advising on different zoonotic diseases during the past 12 months.

### Knowledge of disease symptoms and signs

**Fig 4:**
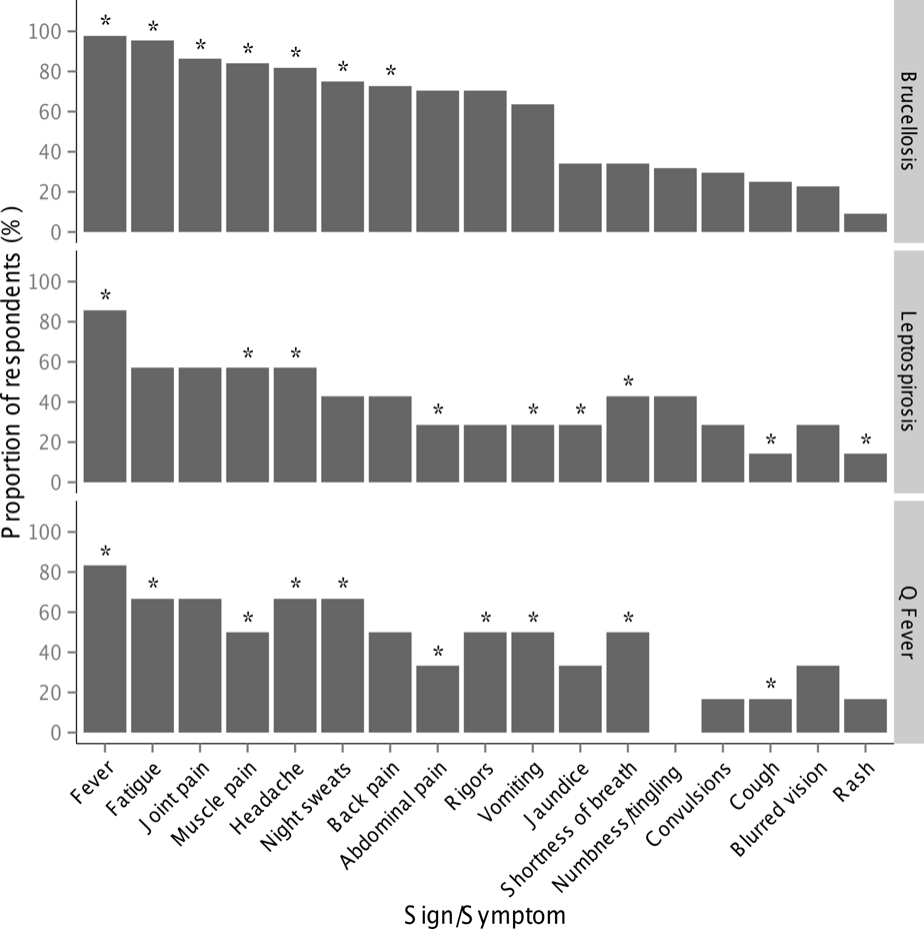
Symptoms and signs of human brucellosis (n = 44), leptospirosis (n = 7), and Q fever (n = 6) reported by healthcare providers in Moshi, Tanzania, 2014–2015. Stars indicate signs and symptoms included in CDC case definitions for each disease. For each disease, all healthcare providers were first asked if they could provide information about the signs and symptoms of the disease in humans. Respondents included representatives of both sectors as follows: 8 animal and 36 human sector respondents for brucellosis, 3 animal and 4 human sector respondents for leptospirosis, one animal and 5 human sector respondents for Q fever.

**Fig 5:**
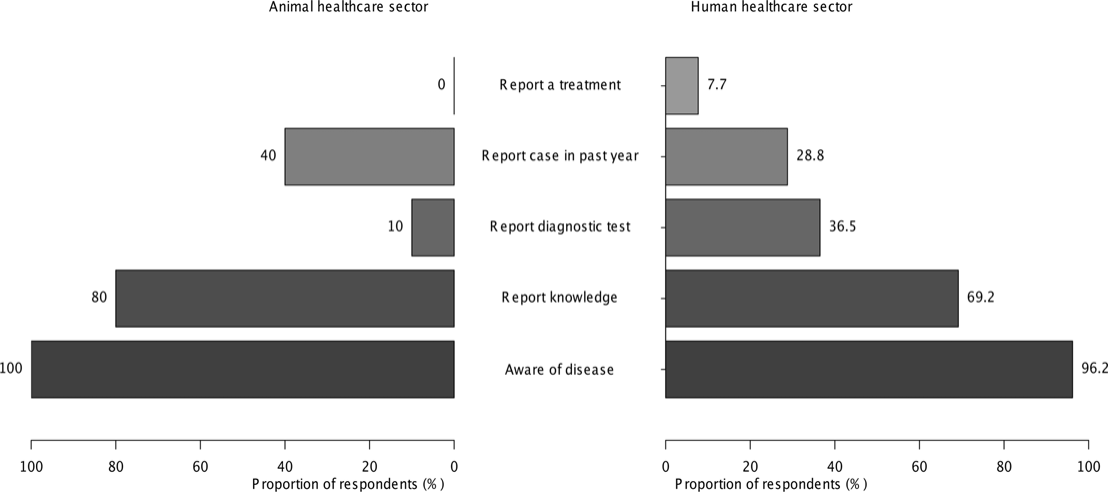
Knowledge and awareness of brucellosis among healthcare providers in Moshi, Tanzania. Proportion of animal (n = 10) and human (n = 52) healthcare providers who reported ‘Yes’ in response to the questions: ‘Have you heard of a disease called brucellosis?’; ‘Can you tell me about the clinical signs and symptoms that are commonly seen with brucellosis in animals/humans?’; ‘Do you advise clients/patients to get a test or can you provide any tests that can be used to diagnose brucellosis in animals/humans?’; and ‘Do you recommend any treatments that can be used to treat brucellosis in animals/humans?’ Figure depicts responses regarding animal brucellosis from animal healthcare providers and responses regarding human brucellosis from human healthcare providers.

Of the 60 respondents reporting awareness of brucellosis, 8 (80%) animal sector respondents and 36 (69%) human sector respondents reported knowledge of its symptoms and signs in humans. Fig 4 summarizes the symptoms and signs that respondents associated with human brucellosis and Fig 5 summarizes knowledge and awareness of brucellosis cases, treatment and testing. The median (range) value of Cohen’s kappa calculated for study respondents in comparison to the CDC brucellosis case definition was 0.39 (0.08 to 0.88). Twenty-two (50%) had Cohen’s kappa values less than 0.4 (slight to fair agreement). Eleven (25%) had values between 0.4 and 0.6 (moderate agreement), and 11 (25%) had values greater than 0.6 (substantial agreement) [32]. None of the respondent characteristics examined, including gender, sector, age, highest educational qualification, or attendance of training after qualification were significantly associated with the respondents’ agreement with the expert classification. All 10 animal sector respondents and 13 (25%) human sector respondents with awareness of brucellosis reported knowledge of brucellosis signs in animals. Of these, 22 (96%) identified abortion, 17 (74%) breeding problems, 17 (74%) retained placenta, 15 (65%) birth of weak offspring, 13 (57%) complete infertility, and 13 (57%) drop in milk production.

Three (38%) animal sector respondents and 4 (22%) human sector respondents aware of leptospirosis reported knowledge of its symptoms and signs in humans, and 1 (20%) animal sector respondent and 5 (33%) human sector respondents reported knowledge of the symptoms and signs of human leptospirosis (Fig 4). None reported knowledge of leptospirosis or Q fever signs in animals.

### Knowledge of diagnostic test options

Of the human healthcare sector respondents aware of brucellosis, 19 (38%) reported awareness of a locally available test for diagnosis of brucellosis in humans. Of these, 8 (42%) reported availability of the test at their facility of employment. One human sector respondent described locally available tests for diagnosis of leptospirosis and Q fever in humans, 1 animal sector respondent described an animal brucellosis diagnostic test, and none described tests for leptospirosis or Q fever in animals.

### Knowledge of disease transmission, prevention, and treatment

Table 2 summarizes respondents’ reported knowledge of brucellosis transmission, prevention, and treatment by sector.

**Table 2:** Reported knowledge of transmission, prevention, and treatment of brucellosis by animal (n = 10) and human (n = 50) sector healthcare providers in Moshi, Tanzania, 2014–2015. Respondents were asked first if they knew about the different categories of brucellosis epidemiology listed below and then to provide examples and details in cases where they reported some knowledge. The table gives the total number of respondents who provided a response in each category and the most frequent responses in each category.

	Respondent sector			
	Livestock		Human health	
	n	(%)	n	(%)
**Knowledge of transmission routes from livestock to humans**	**10**	**(100)**	**47**	**(94)**
Consumption of raw or unpasteurized milk	9	(90)	39	(83)
Consumption of undercooked or infected meat	3	(30)	26	(55)
Direct contact with infected animal products	4	(40)	19	(40)
**Knowledge of transmission routes between livestock**	**8**	**(80)**	**8**	**(16)**
Sexual transmission	7	(88)	1	(13)
Grazing on contaminated pasture	0	(0)	5	(63)
**Knowledge of prevention or treatment of human infection**	**6**	**(60)**	**26**	**(52)**
Boiling milk	5	(83)	19	(73)
Cooking or selecting inspected meat	3	(50)	13	(50)
Specific antimicrobial treatment (doxycycline or streptomycin)	0	(0)	4	(15)
**Knowledge of prevention or treatment of livestock infection**	**9**	**(90)**	**1**	**(2)**
Artificial insemination or controlled livestock breeding practices	4	(44)	0	(0)
Livestock vaccination	3	(33)	1	(100)

One (13%) animal sector respondent and 3 (17%) human sector respondents aware of leptospirosis reported knowledge of routes of leptospirosis transmission to humans, 2 (50%) citing direct contact with infected animal products, 1 (25%) consumption or contact with unpasteurized milk or undercooked meat, and 1 (25%) airborne transmission. No animal sector respondents and 2 (13%) human sector respondents reported knowledge of Q fever transmission to humans; 1 (50%) reported consumption of un-boiled milk or undercooked meat, and 1 (50%) airborne transmission.

No human sector respondents and one (4%) animal sector respondent reported knowledge of leptospirosis transmission among animals, describing contamination of animal feed with rat urine. None reported knowledge of Q fever transmission routes among animals or knowledge of prevention or treatment of leptospirosis or Q fever in humans or animals.

### Semi-structured interviews

Interviews were conducted with 60 respondents. Two respondents declined interview due to reported time constraints. Fifty-nine transcripts were available for analysis due to the loss of one audio recording. Table 1 details the key codes and sub-codes extracted from interview transcripts, including the breakdown of responses by respondent healthcare sector. Indicative quotes from the interviews are included below (with typographic and transcription errors corrected to improve readability). When presented with findings from a previous fever etiology study conducted in the Kilimanjaro Region, 36 (61%) respondents in total indicated that the low malaria prevalence reported by the study was consistent with their expectations (Table 1). Another 20 (34%) indicated that the reported malaria prevalence was lower than they had expected.

When asked about possible explanations for the apparent under-reporting of zoonoses, 44 (75%) respondents indicated that healthcare providers lacked knowledge or awareness of zoonoses. This theme was expanded on in many of the interview responses:

*“I think people just ignore* [referring to a prompt regarding under-diagnosis of zoonoses in the previous etiology study]. *They think it is not possible for people to get diseases from an animal that is why they do not check for those diseases”* (BZQ-009, Human healthcare sector respondent)

*“It is true that most health workers or doctors do not test or diagnose these zoonotic diseases*. *This is because they do not believe that our animals can transmit disease to us*, *therefore many health workers are ignorant of most zoonotic diseases”* (BZQ-032 Animal healthcare sector respondent)

*“It is astonishing news to know that other zoonotic diseases are also found in the area since we don’t have a habit of checking them*. *This is because neither the doctors or health workers not the patients had an idea about them” (*BZQ-064 Animal healthcare sector respondent)

Respondents also indicated that daily experience and the reinforcement (or not) of baseline knowledge also contributed to their consideration (or not) of zoonoses in their daily practice:

*“We have knowledge*, *but sometimes we miss it*. *We have knowledge because we have been taught in school*, *but you find you stay for some years without coming in contact with those diseases*, *until you come to remind me*, *‘Oh*, *I was supposed also to test for diseases transmitted from animals”* (BZQ-018, Human healthcare sector respondent).

*“Today you reminded me what I was taught in school but didn't practice in any working place”* (BZQ-015, Human healthcare sector respondent).

*“If you don't have it in mind*, *you can't test for it”* (BZQ-005, Human healthcare sector respondent).

“*I can’t talk on the knowledge of livestock officers on zoonoses but to be honest—human doctors—we don’t treat zoonotic cases time time*, *so most of us consider as they are not there*.” (BZQ-022, Human healthcare sector respondent)

“*Also there is no awareness on those diseases in hospital*, *that is why you need seminars to remind people because you find people have forgotten*” (BZQ-042 Human healthcare sector respondent)

A lack of testing capacity was also identified as playing a crucial role in the apparent under-diagnosis of zoonoses. Forty-six respondents (78%) pointed to the lack of available tests for zoonoses in the quantitative survey and diagnostic constraints were described explicitly in interviews:

*“People come to the hospital with fever*, *but when you test they don’t have malaria*, *then what do they have*? *You start to guess*, *but if we had facilities to test diseases then you could test and confirm”* (BZQ-037, Human healthcare sector respondent).

Seventeen (29%) respondents mentioned a common assumption among healthcare providers that a fever indicates malaria. Twelve (20%) respondents indicated a lack of confidence in malaria rapid diagnostic tests (mRDTs), and 11 (19%) insisted that sometimes mRDTs produced false negative malaria results when other tests had shown ‘true’ positives. Interview extracts relevant to this theme include:

“*The doctors have developed a habit where they assume people with fever symptoms are suffering from malaria*. *That is why many clinicians do not remember to check zoonotic diseases”* (BZQ-062, Human healthcare sector respondent).

“*I have been working here for a long time and never got malaria positive using the mRDT test*, *though another hospital says they get positive malaria using the mRDT test*. *But I remember in the past while working with the other hospital*, *when mRDT showed negative for malaria*, *I advised them to use the microscope to test and the results would be positive for malaria*, *so I think people should not only rely on the mRDT test”* (BZQ-030, Human healthcare sector respondent).

## Discussion

We conducted this study to explore possible barriers to the recognition and reporting of zoonoses in Moshi, Tanzania. Our findings reveal several considerable obstacles to the diagnosis of zoonoses cases, including under-appreciation of zoonoses in differential diagnoses, deficiencies in diagnostic test capacities, and persistent pressures that lead to the relative over-diagnosis of malaria and under-diagnosis of other causes of fever including many zoonoses.

Brucellosis, leptospirosis, and Q fever all cause febrile illness among people in the Moshi area and are also likely to cause livestock productivity losses [9]. In this study population, knowledge and awareness of brucellosis was consistently higher than for leptospirosis and Q fever. Many respondents were able to accurately report symptoms and signs, routes of transmission, and disease prevention strategies for brucellosis in both animals and humans. For topics relating to the epidemiology of brucellosis in humans, similar proportions of respondents from the human and animal sectors reported knowledge and were able to provide descriptions of the epidemiology of this disease. For topics relating specifically to livestock however this was not the case and respondents from the human healthcare sector were much less likely to report knowledge (Table 2). For leptospirosis and Q fever, awareness was much lower and this difference was more marked for respondents from the human healthcare sector (Fig 2). Brucellosis cases were reported by respondents from both the animal and human healthcare sectors, but no cases of leptospirosis or Q fever were described (Fig 3).

Lack of knowledge of zoonoses among healthcare providers was commonly highlighted as a probable contributor to under-reporting during interviews. The data presented here reveal higher levels of knowledge of brucellosis as compared to leptospirosis and Q fever as well as more frequent case reports in both animal and human healthcare sectors. This suggests a clear and intuitive link between the relative knowledge of different diseases and the frequency of recognition in daily practice. The true local prevalence of a disease is also likely to impact on levels of familiarity. However, previous work in the Moshi area has revealed lower prevalence of acute brucellosis than either leptospirosis and Q fever in the human febrile patient population. Differences in training and exposure to research or public health education projects and the overall prioritization and profile of different diseases are also likely contribute to differences in the awareness and knowledge of diseases amongst community level healthcare providers.

The quantitative survey findings regarding brucellosis particularly suggest that under-recognition of zoonoses may not be driven entirely by a lack of awareness or knowledge *per se*. In the interviews, several human healthcare respondents identified a mismatch between their background knowledge or awareness and their consideration of zoonoses in their daily practice, suggesting that both knowledge in the first instance and capacity to relate that knowledge to daily experience are important. The above quotations (respondents BZQ-018, 015 and 005) reveal the crucial significance of reinforcement of knowledge through regular recognition and experience of zoonoses cases to ensure that these pathogens are kept in mind. For healthcare providers to recognize and experience zoonoses in their daily practice, it must be possible either to diagnose a disease based on clinical presentation, to perform an accurate diagnostic test or to document patient recovery after giving a specific treatment. When asked to name zoonoses transmitted from livestock to humans, many respondents named zoonoses that can be described as high visibility and high impact, with clinically distinctive and dramatic presentations (e.g., anthrax, rabies, and Ebola virus disease). In contrast, the three bacterial zoonoses of key interest in this study have non-specific manifestations in both humans and animals, greatly complicating recognition and contributing to their apparent on-going invisibility to healthcare providers.

The frequency of reports of available diagnostic tests for brucellosis, leptospirosis, and Q fever were consistently low and a lack of resources for tests for zoonoses was identified as an important factor influencing zoonoses under-reporting by high proportions of both livestock and human sector interview respondents. A variety of serological tests for brucellosis are available in northern Tanzania. However, access to these tests is not uniform, and concerns have been raised about the performance of many of these diagnostics. One survey respondent indicated that tests for leptospirosis and Q fever in humans were available, but we are not aware of any widely accessible tests for these pathogens in the study area. Given the challenges of clinically diagnosing brucellosis, leptospirosis, or Q fever in both animals and humans, it is therefore unsurprising that few respondents reported seeing cases within the past year [33,34].

The low frequency of zoonoses diagnoses also appears to be tied to persistent perceptions that malaria is the primary etiology of fever, even though epidemiological evidence increasingly contradicts this notion [9,16]. Although the numbers are small, it is revealing that healthcare providers from the livestock sector were twice as likely to mention the view that human healthcare providers believe that fever and malaria are synonymous and the influence of this view upon patient care, than were their human healthcare sector equivalents. As compared to livestock sector healthcare providers, greater proportions of respondents from the human healthcare sector indicated that the prevalences of zoonoses reported in the Crump et al study [9] were higher than they would have expected.

Several interview responses about the use and interpretation of mRDTs suggest that the existence of the technological diagnostic capacity is necessary but probably not sufficient to lead to desired improvements in zoonoses diagnosis and treatment. As reported in studies from other sites in Africa [35,36], our study interviews revealed a lack of confidence in negative mRDT results amongst human healthcare providers. A conclusion that more and better diagnostic tests for zoonoses would be valuable is not novel. However, relatively few field-validated diagnostics are available for the accurate diagnosis of acute zoonoses and this undoubtedly contributes to the persistent under-reporting of many zoonoses. The demonstration of seroconversion in paired acute and convalescent phase sera remains central to the confirmed diagnosis of brucellosis, leptospirosis and Q fever in acute human cases. This testing approach is invaluable for generating epidemiological data but is not a practical option for clinicians managing acutely ill patients. Work to develop improved diagnostics is ongoing. Additionally, work to raise awareness and knowledge of zoonoses in endemic settings may help to reduce under-diagnosis even in the absence of locally available diagnostics. When diagnostic test capacity is limited, syndrome-based approaches to patient classification and management can be useful tools. Further studies to inform the management of febrile patients and develop empiric treatment guidelines for physicians are therefore also needed [37].

More fundamentally, investment in animal infection control strategies is necessary to reduce the multiple impacts of zoonoses on human health, animal health, and household livelihoods. The lack of integration between animal and human healthcare sectors is frequently identified as a key barrier to the uptake of One Health strategies, such as animal vaccination campaigns, as a means of reducing health impacts in both animals and people. The data presented here reveal awareness in both human and animal healthcare sector workers of key aspects of zoonoses epidemiology (particularly regarding brucellosis), including host-pathogen relationships, transmission routes, and disease prevention and treatment. Overall, the data presented here indicate a greater degree of cross-disciplinary awareness and knowledge amongst livestock sector healthcare providers than amongst their human sector counterparts, suggesting a greater need for further awareness raising and training for human healthcare providers specifically.

A limitation of this study was its restriction to formal sector healthcare workers in an urban setting, which may not be representative of knowledge and practices in the informal healthcare sector or rural communities. Additionally, healthcare seeking behavior by individuals with zoonotic infection and their understanding of the causes of illness are important contributors to the diagnostic process that were not explored.

This study has demonstrated heterogeneous awareness and knowledge and infrequent case recognition of different zoonoses among healthcare providers in Moshi, Tanzania, an area of zoonotic disease endemicity. Moreover, it has identified several key barriers to zoonoses recognition and reporting, including limitations in diagnostic testing capacity and ingrained perceptions that febrile illness is primarily caused by malaria. By using a mixed methods approach, we have been able to complement questionnaire data characterizing awareness and knowledge of zoonoses with an understanding of the perceptions and perspectives of healthcare providers that drive zoonoses under-diagnosis and malaria over-diagnosis while engaging study participants in a crucial but neglected dialogue underscoring the importance of zoonoses. The findings reveal how deficiencies in diagnostic testing services and the non-specific presentation of many zoonoses can lead to failure to confirm diagnosis as well as a failure to reinforce awareness of cases through the practical experience of diagnosing and treating them, thus leading to a failure to recognize and diagnose in the future. Our study extends existing knowledge by exploring existing awareness and capacity at multiple steps in the processes required for the recognition, treatment and reporting of zoonoses cases. We also contextualize our findings using the qualitative data generated, revealing the need for attention to be paid to the ways that new diagnostic tests are introduced in order to ensure that they are appropriately and usefully integrated into clinical practice. This goal is central to future improvements in the diagnosis, treatment, and reporting of zoonotic diseases here and in other low-resource endemic settings.

## Supporting Information

S1 Questionnaire Questionnaire used for study. (PDF)

S1 Interview Schedule Interview schedule used for study. (PDF)
