# Supplementary material for: Mixed Methods Survey of Zoonotic Disease Awareness and Practice among Animal and Human Healthcare Providers in Moshi, Tanzania
Source: PLoS Negl Trop Dis. 2016 Mar 4;10(3):e0004476. doi: 10.1371/journal.pntd.0004476 (PMC4778930; doi:10.1371/journal.pntd.0004476)
Supplement: S1 Questionnaire — (PDF) [file pntd.0004476.s001.pdf]

2239291413

BACTERIAL ZOONOSES - HEALTHCARE PROVIDER QUESTIONNAIRE

SECTION 1: INTERVIEW DETAILS

Questionnaire ID

BZQ - ☐☐☐

Review Date (dd/mm/yyyy)

☐☐ / ☐☐ / ☐☐☐☐

Interview Date (dd/mm/yyyy)

☐☐ / ☐☐ / ☐☐☐☐

Interviewer's initials

☐☐☐

Reviewer's initials

☐☐☐

Language

O Kiswahili O English O Maasai

SECTION 2: RESPONDENT DETAILS

Sex

O Male O Female

Date of birth (dd/mm/yyyy)

☐☐ / ☐☐ / ☐☐☐☐

If only the year of birth is known, record 01 for dd and 07 for mm.

If year of birth is known, go to next question

Age Class

O 0-5 yr O 6-12 yr O 13-18 yr O 19-34 yr O 35-54 yr O ≥55 yr

How many years of education have you had?

Umepata elimu (darasani) kwa miaka mingapi?

O No education (Sijasoma)
O Primary (1-7 years) (Msingi)
O Secondary (8-11 years) (Sekondari)
O High school (12-13 years) (Sekondari ya juu)
O University/college (Chuo kikuu/chuo)

In what year did you complete your highest level of education?

Ni mwaka gani ulimaliza elimu yako ya juu?

☐☐☐☐

At which institution did you achieve this highest qualification?

Elimu yako ya juu ulimaliza katika chuo/taasisi gani?

Please describe your current job

Tafadhali eleza nafasi yako ya kazi ya wakati huu

Name of workplace

Location of workplace

Region O Kilimanjaro O Arusha

District

O Arumeru O Moshi urban
O Arusha O Monduli
O Longido O Mwanga
O Moshi rural O Rombo

Ward

☐☐☐☐☐☐☐☐☐☐☐☐☐☐

Village/Mtaa

☐☐☐☐☐☐☐☐☐☐☐☐☐☐

Job title

Text

Employer

Employer Classification

☐☐

Employment Duration

Freeform

Months

☐☐☐

TH = tertiary hospital
RH = regional hospital
DH = district hospital
MH = mission hospitall
HC = health centre
PH = private clinic (human)
PA = private clinic (animal)
UC = university/college
LG = local govt.
SE = self

SECTION 3:TRAINING

Since obtaining your highest qualification have you done any training? O Yes O No

Tangu upate elimu yako ya juu kabisa umeshapata mafunzo yoyote ya elimu ya kujiendeleza kitaaluma?

If yes, please gives details of any training attended in the past 12 months.

Kama ndio, tafadhali eleza kwa undani mpango wowote wa mafunzo uliohudhuria katika miezi 12 iliyopita

Topic and description of courses attended

Organisation(s) providing training

Are you a member of any professional organisations?

Je wewe hi mwanachama wa chama chochote cha kitaaluma?

O Yes O No

If yes, please give details of any professional organisations you belong to.

Kama ndio tafadhali toa maelezo ya chama chochote cha kitaaluma ambacho wewe ni mwanachama wake na lini ulijiunga

Name of organisation

Description of organisation

Do you provide services for the animal sector, human or both?

Unatoa huduma katika kitengo cha wanyama, binadamu au vyote?

O Animal O Human O Both

☐☐☐☐☐☐

SECTION 4: GENERAL ZOONOSES KNOWLEDGE

Do you know of any diseases that people can catch from livestock?
Je, unayajua magonjwa yoyote ambayo watu wanaweza kupata kutoka kwa mifugo?

If yes, record the names of the diseases mentioned. O Yes O No

Do you know of any diseases that people can catch from rodents?
Je, unayajua magonjwa yoyote ambayo watu wanaweza kupata kutoka kwa panya?

If yes, record the names of the diseases mentioned. O Yes O No

Do you know of any diseases that people can catch from dogs?
Je, unayajua magonjwa yoyote ambayo watu wanaweza kupata kutoka kwa mbwa?

If yes, record the names of the diseases mentioned. O Yes O No

Do you know of any diseases that can cause abortions in livestock?
Unafahamu magonjwa yoyote ambayo yanaweza kusababisha mimba kuharibika kwa mifugo?

If yes, record the name of the diseases mentioned. O Yes O No

Have you heard of a disease called brucellosis?
Je, umewahi kusikia ugonjwa unaoitwa brucellosis/brusela?

O Yes O No

If no, go to next question

Do you know if this disease (brucellosis) affects only animals, only people or both?
Je unafahamu kuwa ugonjwa huu (brusela) unaathiri wanyama peke yake, watu tu, au wote?

O Only animals (wanyama tu)
O Only humans (binadamu tu)
O Both (wote)

Have you heard of a disease called leptospirosis?
Je, umewahi kusikia ugonjwa unaoitwa leptospirosis?

O Yes O No

If no, go to next question

Do you know if this disease (leptospirosis) affects only animals, only people or both?
Je unafahamu kuwa ugonjwa huu (leptospirosis) unaathiri wanyama peke yake, watu tu, au wote?

O Only animals (wanyama tu)
O Only humans (binadamu tu)
O Both (wote)

Have you heard of a disease called Q fever?
Je, umewahi kusikia ugonjwa unaoitwa Q fever?

O Yes O No

If no, go to next question

Do you know if this disease (Q fever) affects only animals, only people or both?
Je unafahamu kuwa ugonjwa huu (Q fever) unaathiri wanyama peke yake, watu tu, au wote?

O Only animals (wanyama tu)
O Only humans (binadamu tu)
O Both (wote)

Have you heard of a disease called typhoid fever?
Je, umewahi kusikia ugonjwa unaoitwa typhoid fever? O Yes O No

If no, go to next question

Do you know if this disease (typhoid fever) affects only animals, only people or both?
Je unafahamu kuwa ugonjwa huu (typhoid fever) unaathiri wanyama peke yake, watu tu, au wote?

O Only animals (wanyama tu)
O Only humans (binadamu tu)
O Both (wote)

Have you heard of a disease called histoplasmosis?
Je, umewahi kusikia ugonjwa unaoitwa histoplasmosis? O Yes O No

If no, go to next question

Do you know if this disease (histoplasmosis) affects only animals, only people or both?
Je unafahamu kuwa ugonjwa huu (histoplasmosis) unaathiri wanyama peke yake, watu tu, au wote?

O Only animals (wanyama tu)
O Only humans (binadamu tu)
O Both (wote)

1067291411

SECTION 5: ZOONOSES REPORTED BY RESPONDENT

We are interested in finding out what zoonotic diseases (diseases that people can catch from animals) are present in this area.
Tunapenda kufahamu magonjwa ya wanyama na binadamu (magonjwa ambayo watu wanaweza kupata kutako kwa wanyama) yaliyopo katika eneo hili.

Please give the names and details of all of the zoonotic diseases that you have seen or advised on during the past 12 months through your work.
Tafadhali taja majina na maelezo ya magonjwa yote ya wanyama na binadamu ambayo umeona au kutolea ushauri katika kipindi cha miezi 12 iliyopita kupitia kazi yako.

Please complete a new row of the table below for each disease that you have seen. For each row please record:
the name of the disease the species affected
the signs that led to diagnosis/ description of the disease signs
the details of any tests /procedures used to reach a diagnosis (including clinical evaluations)
the treatment or recommendation and any additional advice that you provided

After the respondent has listed the zoonoses that they have seen voluntarily please prompt specifically to find out about any cases that they have seen in the past 12 months of the following diseases: brucellosis; plague; leptospirosis; anthrax; Q fever; rabies; trypanosomiasis
If details of any cases are reported, record "Yes" for "Any zoonoses". If no cases are reported, record "No" .

Any zoonoses? ○ Yes ○ No

Brucellosis? ○ Yes ○ No

Plague / Tauni? ○ Yes ○ No

Leptospirosis? ○ Yes ○ No

Anthrax / Kimeta? ○ Yes ○ No

Q fever ○ Yes ○ No

Rabies? ○ Yes ○ No

Trypanosomiasis / Malale? ○ Yes ○ No

Name	Species Prompted?	Description/ Signs	Tests	Treatment/Management
	○ V ○ P			
	○ V ○ P			
	○ V ○ P			
	○ V ○ P			
	○ V ○ P			

Cattle = BO; Sheep = OV; Goats = CP; Pigs = PO; Dogs = CA; Cats = FE Humans = HU
If other write name in full.

SECTION 6: SIGNS & SYMPTOMS IN HUMANS

Can you tell me about the clinical signs and symptoms that are commonly seen with the following diseases in humans?
Je unaweza kuniambia kuhusu dalili au viashiria vya kitibabu ambavyo vinaonekana kwa kawaida kwa magonjwa yafuatayo kwa binadamu?

Brucellosis O Yes O No **Q Fever** O Yes O No

Leptospirosis O Yes O No **Rabies** O Yes O No

If No for a specific disease, do not complete the relevant box or table sections below.
First ask the respondent to tell you the signs and symptoms that they know and record these volunteered responses (V) in the table.
Then go through the list of symptoms/signs and prompt the respondent to find out if they think each is associated with the disease(s) listed. Record a Yes (Y) or No (N) response after prompting. Record any additional reported signs or symptoms in the text boxes.

	Brucellosis	Leptospirosis	Q Fever	Rabies
joint pain<br>**maumivu ya viungo**	O V O Y O N	O V O Y O N	O V O Y O N	O V O Y O N
shortness of breath<br>**kupumua kwa shida**	O V O Y O N	O V O Y O N	O V O Y O N	O V O Y O N
blurred vision<br>**kuona kwa shida**	O V O Y O N	O V O Y O N	O V O Y O N	O V O Y O N
numbness/tingling of extremities<br>**ganzi**	O V O Y O N	O V O Y O N	O V O Y O N	O V O Y O N
cough<br>**kohoa**	O V O Y O N	O V O Y O N	O V O Y O N	O V O Y O N
abdominal pain<br>**maumivu ya tumbo**	O V O Y O N	O V O Y O N	O V O Y O N	O V O Y O N
headache<br>**maumivu ya kichwa**	O V O Y O N	O V O Y O N	O V O Y O N	O V O Y O N
convulsions<br>**degedege**	O V O Y O N	O V O Y O N	O V O Y O N	O V O Y O N
rigors<br>**kuteteimeka baridi**	O V O Y O N	O V O Y O N	O V O Y O N	O V O Y O N
night sweats<br>**kutokwa na jasho usiku**	O V O Y O N	O V O Y O N	O V O Y O N	O V O Y O N
fever<br>**homa**	O V O Y O N	O V O Y O N	O V O Y O N	O V O Y O N
jaundice/yellow eyes<br>**manjano/macho ya njano**	O V O Y O N	O V O Y O N	O V O Y O N	O V O Y O N
rash<br>**kovu**	O V O Y O N	O V O Y O N	O V O Y O N	O V O Y O N
fatigue<br>**kuchoka mwili**	O V O Y O N	O V O Y O N	O V O Y O N	O V O Y O N
muscle pain<br>**maumivua ya misuli**	O V O Y O N	O V O Y O N	O V O Y O N	O V O Y O N
back pain<br>**maumivu ya mgongo**	O V O Y O N	O V O Y O N	O V O Y O N	O V O Y O N
vomiting<br>**kutapika**	O V O Y O N	O V O Y O N	O V O Y O N	O V O Y O N

Brucellosis

Q Fever

Leptospirosis

Rabies

SECTION 7: SIGNS IN ANIMALS

Can you tell me about the clinical signs that are commonly seen with the following diseases in animals?
Unaweza kuniambia kuhusu dalili za kitibabu ambavyo zinazoonekanakwa kawaida kwa magonjwa yafuatayo kwa wanyama?

Brucellosis O Yes O No **Q Fever** O Yes O No

Leptospirosis O Yes O No **Anthrax** O Yes O No

If No for a specific disease, do not complete the relevant box or table section below.
First ask the respondent to tell you the signs that they know and record these volunteered responses (V) in the table.
Then go through the list of signs and prompt the respondent to find out if they think each is associated with the disease(s) listed. Record a Yes (Y) or No (N) response after prompting. Record any additional reported signs in the text box on the next page. For each sign that is recorded (V&Y responses), record which species this sign is seen in.

	Brucellosis	Leptospirosis	Q Fever	Anthrax
swollen testicles<br>***kende kuvimba***	O V O BO<br>O Y O CP<br>O N O OV	O V O BO<br>O Y O CP<br>O N O OV	O V O BO<br>O Y O CP<br>O N O OV	O V O BO<br>O Y O CP<br>O N O OV
complete infertility<br>***tasa kabisa***	O V O BO<br>O Y O CP<br>O N O OV	O V O BO<br>O Y O CP<br>O N O OV	O V O BO<br>O Y O CP<br>O N O OV	O V O BO<br>O Y O CP<br>O N O OV
breeding (conception and gestation) problems<br>***matatizo ya kuzaa (kutopata mimba au mimba kutokukua)***	O V O BO<br>O Y O CP<br>O N O OV	O V O BO<br>O Y O CP<br>O N O OV	O V O BO<br>O Y O CP<br>O N O OV	O V O BO<br>O Y O CP<br>O N O OV
mastitis (including changes in the milk e.g. blood, clots of pus etc.)<br>***ugonjwa wa kiwele (pamoja na mabadiliko ya maziwa mfano damu, usaha n.k.)***	O V O BO<br>O Y O CP<br>O N O OV	O V O BO<br>O Y O CP<br>O N O OV	O V O BO<br>O Y O CP<br>O N O OV	O V O BO<br>O Y O CP<br>O N O OV
unexplained drop in mik production (i.e. not attributed to e.g. reduced diet)<br>***kupungua utoaji maziwa kusikoelezeka (mfano, hakuhusiani na kupungua chakula)***	O V O BO<br>O Y O CP<br>O N O OV	O V O BO<br>O Y O CP<br>O N O OV	O V O BO<br>O Y O CP<br>O N O OV	O V O BO<br>O Y O CP<br>O N O OV
mouth lesions<br>**vidonda mdomoni**	O V O BO<br>O Y O CP<br>O N O OV	O V O BO<br>O Y O CP<br>O N O OV	O V O BO<br>O Y O CP<br>O N O OV	O V O BO<br>O Y O CP<br>O N O OV
retained placenta<br>**kubaki kwa kondo la nyuma**	O V O BO<br>O Y O CP<br>O N O OV	O V O BO<br>O Y O CP<br>O N O OV	O V O BO<br>O Y O CP<br>O N O OV	O V O BO<br>O Y O CP<br>O N O OV
joint swelling<br>***kuvimba viungo***	O V O BO<br>O Y O CP<br>O N O OV	O V O BO<br>O Y O CP<br>O N O OV	O V O BO<br>O Y O CP<br>O N O OV	O V O BO<br>O Y O CP<br>O N O OV
lameness<br>***udhaifu wa miguu***	O V O BO<br>O Y O CP<br>O N O OV	O V O BO<br>O Y O CP<br>O N O OV	O V O BO<br>O Y O CP<br>O N O OV	O V O BO<br>O Y O CP<br>O N O OV
birth of weak offspring<br>***kuzao ndama mdhaifu***	O V O BO<br>O Y O CP<br>O N O OV	O V O BO<br>O Y O CP<br>O N O OV	O V O BO<br>O Y O CP<br>O N O OV	O V O BO<br>O Y O CP<br>O N O OV
incoordination or paralysis<br>***kupooza au matatizo ya viungo kuwasiliana***	O V O BO<br>O Y O CP<br>O N O OV	O V O BO<br>O Y O CP<br>O N O OV	O V O BO<br>O Y O CP<br>O N O OV	O V O BO<br>O Y O CP<br>O N O OV
bloody urine<br>**mkojo wa damu**	O V O BO<br>O Y O CP<br>O N O OV	O V O BO<br>O Y O CP<br>O N O OV	O V O BO<br>O Y O CP<br>O N O OV	O V O BO<br>O Y O CP<br>O N O OV
abortion or stillbirth<br>***kutapa mimba au kuzaa ndama mfu***	O V O BO<br>O Y O CP<br>O N O OV	O V O BO<br>O Y O CP<br>O N O OV	O V O BO<br>O Y O CP<br>O N O OV	O V O BO<br>O Y O CP<br>O N O OV
anaemia (e.g. pallor and pale membranes)<br>***kupungua damu (weupe na weupe kwenye ngozi laini)***	O V O BO<br>O Y O CP<br>O N O OV	O V O BO<br>O Y O CP<br>O N O OV	O V O BO<br>O Y O CP<br>O N O OV	O V O BO<br>O Y O CP<br>O N O OV
jaundice (e.g. yellow eyes)<br>***manjano (mfano, macho kuwa manjano)***	O V O BO<br>O Y O CP<br>O N O OV	O V O BO<br>O Y O CP<br>O N O OV	O V O BO<br>O Y O CP<br>O N O OV	O V O BO<br>O Y O CP<br>O N O OV
bleeding from the nose or gums<br>***kutokwa na damu puani au kwenye fizi***	O V O BO<br>O Y O CP<br>O N O OV	O V O BO<br>O Y O CP<br>O N O OV	O V O BO<br>O Y O CP<br>O N O OV	O V O BO<br>O Y O CP<br>O N O OV

Record any additional reported signs for any of the four diseases in animals in the text box below (recording the disease and species names).

SECTION 8: TRANSMISSION

Do you know of any of the ways that people can become infected with each of the following diseases?
Je unafahamu njia yoyote ambayo binadamu anaweza kuambukizwa kwa kila aina ya magonjwa yafuatayo mfano?
If yes, record details below

Brucellosis O Yes O No
Leptospirosis O Yes O No
Q Fever O Yes O No

Brucellosis - Human Transmission

Leptospirosis - Human Transmission

Q Fever - Human Transmission

Do you know of any of the ways that animals can become infected with each of the following diseases?
Je unafahamu njia yoyote ambayo wanyama wanaweza kuja kuambukizwa kwa kila aina ya magonjwa yafuatayo?
If yes, record details below

Brucellosis O Yes O No
Leptospirosis O Yes O No
Q Fever O Yes O No

Brucellosis - Animal Transmission

Leptospirosis - Animal Transmission

Q Fever - Animal Transmission

SECTION 9: TESTING

Do you advise customers/clients to get a test or can you provide any tests that can be used to diagnose the following diseases in animals?
Je huwa unawashauri wateja kwenda kufanya vipimo au unaweza kufanya vipima ambavyo vinaweza kutumikakutambua magonjwa yafuatayo kwa wanyama?

Brucellosis O Yes O No
Leptospirosis O Yes O No
Q Fever O Yes O No

If No for a specific disease, do not complete the relevant box or table section below.
Complete a new row of the table for each disease and test combination you advise/ have access to and for that test record the approx time since you last advised /referred a client to get tested or used that test.

Disease	Diagnostic tests or procedures used	Test location	Location if Other? *(blank if Location = Here)*	When last used/advised? *Freeform*	*Months before*
O B O L O Q		O Here O Other			
O B O L O Q		O Here O Other			
O B O L O Q		O Here O Other			

Do you advise patients to get a test or can you provide tests that can be used to diagnose the following diseases in humans?
Je huwa unashauri wagonjwa kwenda kufanya vipimo au unaweza kufanya vipimo ambauyo vinaweza kutumika kutambua magonjwa yafuatayo kwa binadamu?

**Brucellosis**	O Yes	O No
**Leptospirosis**	O Yes	O No
**Q Fever**	O Yes	O No

If No for a specific disease, do not complete the relevant box or table section below.
Complete a new row of the table for each disease and test combination you advise/ have access to and for that test record the approx time since you last advised /referred a patient to get tested or used that test.

Disease	Diagnostic tests or procedures used	Test location	Location if Other? *(blank if Location = Here)*	When last used/advised? *Freeform*	*Months before*
O B O L O Q		O Here O Other			
O B O L O Q		O Here O Other			
O B O L O Q		O Here O Other			

SECTION 10: PREVENTION & TREATMENT

Do you recommend any preventions or treatments that can be used to prevent or treat the following diseases in humans?
Je huwa unapendekeza kinga au matatibu yeyote ambayo yanaweza kutumika kukinga au kutibu magonjwa yafuatayo kwa binadamu?

**Brucellosis**	O Yes	O No
**Leptospirosis**	O Yes	O No
**Q Fever**	O Yes	O No

If Yes for a specific disease please record the details of any treatments (including drug names, dosage, frequency and duration of treatment in the appropriate box below.

Brucellosis - Human Prevention & Treatment	Leptospirosis - Human Prevention & Transmission	Q Fever - Human Prevention & Transmission

Do you recommend any preventions or treatments that can be used to prevent or treat the following diseases in animals?
Je huwa unapendekeza kinga au matibabu yeyote ambayo yanaueza kutumika kukinga au kutibu magonjwa yafuatayo kwa wanyama?

**Brucellosis**	O Yes	O No
**Leptospirosis**	O Yes	O No
**Q Fever**	O Yes	O No

If Yes for a specific disease please record the details of any treatments (including drug names, dosage, frequency and duration of treatment in the appropriate box below.

Brucellosis - Animal Transmission	Leptospirosis - Animal Transmission	Q Fever - Animal Transmission

SECTION 11: COMMENTS & FEEDBACK

Please record the key points of any discussion/ follow-up conversation **that you have with the respondent after talking through the study feedback.**

Points to record notes on should include:

- How much discussion/ interest there was?

- Did the respondent provide any answers/ feedback in response to the questions listed at the end of the feedback sheet and if so what?

- Did the respondent ask any questions about the interview process, feedback, project etc?

- What were the questions? And what did you say to answer?
