# Supplementary material for: Mixed Methods Survey of Zoonotic Disease Awareness and Practice among Animal and Human Healthcare Providers in Moshi, Tanzania
Source: PLoS Negl Trop Dis. 2016 Mar 4;10(3):e0004476. doi: 10.1371/journal.pntd.0004476 (PMC4778930; doi:10.1371/journal.pntd.0004476)
Supplement: S1 Interview Schedule — (PDF) [file pntd.0004476.s002.pdf]

BACTERIAL ZOONOSES PROJECT

Kilimanjaro Christian Medical Centre, Sokoine University of Agriculture, University of Glasgow & Duke University Research Collaboration

Our project is focused on bacterial zoonoses – bacterial diseases that are transmitted between animals and people. The diseases that we are most interested in are brucellosis, leptospirosis and Q fever which all cause fever in people in this area and are carried by livestock species.

Our study uses several disciplinary approaches, including socio-economic and behavioural studies, human febrile illness surveillance, and linked human-animal epidemiological studies to learn more about the knowledge, risks and impacts of bacterial zoonotic diseases in peri-urban, agropastoral and pastoral settings in northern Tanzania.

We are conducting the survey that you have participated in to learn more about the experience and awareness of zoonoses that different animal and human healthcare providers working in northern Tanzania have.

A recent study that was carried out at KCMC and MRH in Moshi in 2007-8 looked at the range of diseases that cause fever in people from this area. This study found that:

- For every 100 febrile admissions, malaria was the clinical diagnosis for roughly 61 patients but was the actual cause of fever in only 1 or 2 our of 100.
- In contrast, acute bacterial zoonoses were identified among approximately a quarter of febrile admissions – roughly one in four people with fever had a bacterial zoonosis
- For every 100 patients admitted with fever roughly 4 patients had brucellosis
- For every 100 patients admitted with fever roughly 9 patients had leptospirosis
- For every 100 patients admitted with fever roughly 5 patients had Q fever
- For every 100 patients admitted with fever roughly 8 patients had spotted fever group rickettsioses
- For every 100 patients admitted with fever roughly 1 patients had typhus group rickettsioses
- None of the patients with bacterial zoonoses had a bacterial zoonosis included in the admission differential diagnosis

We are interested in your feedback on these findings:

- Is the number of patients with fever who have malaria higher or lower than you would have thought?
- Are the numbers of patients with fever who have bacterial zoonoses higher or lower than you would have thought?
- Why do you think that none of the patients in this previous study were initially diagnosed with any of these bacterial zoonoses?
- Do you have any comments or questions about these findings?

Many thanks for your participation in our study.

BACTERIAL ZOONOSES PROJECT

Kilimanjaro Christian Medical Centre, Sokoine University of Agriculture, University of Glasgow & Duke University Research Collaboration

Mradi wetu unahusiana na magonjwa kati ya wanyama na binadamu yanayoambukizwa na bakteria. Magonjwa ambayo tunayoyaangalia zaidi ni brucellosis, leptospirosis and Q fever ambayo yote yanasabisha homa kwa binadamu katika eneo hili na vimelea vya magonjwa haya vipo ndani ya mifugo.

Mradi wetu unajumisha njia mbalimbali za kitafiti, zikiwemo uchumi-jamii (socio-economic) na mwenendo wa kitabia, uchunguzi wa homa za binadamu na kujifunza muingiliano wa magonjwa kati ya binadamu na wanyama ili kujifunza zaidi ili kupata uelewa, athari na madhara ya magonjwa yanayoambukizwa kutoka kwa wanyama kwenda kwa binadamu yatokanayo na bacteria kwenye maeneo yanayokaribiana na mji, maeneo ya wakulima-wafugaji na maeneo ya wafugaji pekee kaskazini mwa Tanzania.

Tunafanya utafiti ambao wewe umeshiriki ili kujifunza zaidi kuhusiana na uzoefu na uelewa wa magonjwa ambayo binadamu anapata kutoka kwa wanyama ambayo watoa huduma tofauti wa wanyama na binadamu kaskazini mwa Tanzania wanayo.

Utafiti wa hivi karibuni uliofanyika katika hospitali za KCMC na Mawenzi zilizopo Moshi mwaka 2007-2008 waliangalia magonjwa tofauti tofauti ambayo yanasababisha homa kwa binadamu katika eneo hili. Utafiti huo uligundua yafuatayo:

- Kwa kila wagonjwa 100 waliofika hospitali wakiwa na homa, kwa kuangalia dalili waliogundulika na malaria, walikadiriwa wagonjwa 61 lakini waliokuwa na homa kweli iliyosababishwa na malaria ni mgonjwa 1 au 2 kati ya 100.
- Kwa upande mwingine, magonjwa yanayoambukizwa kwa haraka kutoka kwa wanyama kwenda kwa binadamu yasababishwayo na bakteria yaligundulika takribani robo ya wagonjwa wenye homa waliofika hospitali – kwa kukadiria mtu mmoja kati ya wagonjwa wanne wenye homa alikuwa na magonjwa yanayoambukizwa kutoka kwa wanyama kwenda kwa binadamu yasababishwayo na bakteria.
- Kwa kila wagonjwa 100 waliolazwa wakiwa na homa, kwa makadirio wagonjwa 4 walikuwa na brucellosis.
- Kwa kila wagonjwa 100 waliolazwa wakiwa na homa, kwa makadirio wagonjwa 9 walikuwa na leptospirosis.
- Kwa kila wagonjwa 100 waliolazwa wakiwa na homa, kwa makadirio wagonjwa 5 walikuwa na Q fever.
- Kwa kila wagonjwa 100 waliolazwa wakiwa na homa, kwa makadirio wagonjwa 8 walikuwa na spotted fever group rickettsioses.

- Kwa kila wagonjwa 100 waliolazwa wakiwa na homa, kwa makadirio wagonjwa 1 walikuwa na typhus group rickettsioses.
- Hakuna wagonjwa wenye magonjwa yasababishwayo na bacteria kutoka kwa wanyama kwenda kwa binadamu ambao yaliyofikiriwa kuwepo kwa mgonjwa kati ya magonjwa yanayosababisha homa pindi alipokuwa akilazwa

Tunapendelea mrejesho wako kulingana na matokeo hayo:

- Unafikiri idadi ya wagonjwa wenye homa ambao wana malaria ni kubwa au ndogo?
- Unafikiri idadi ya wagonjwa wenye homa ambao wana magonjwa yasababishwayo na bacteria kutoka kwa wanyama kwenda kwa binadamu ni kubwa au ndogo?
- Unafikiri ni kwa nini hakuna wagonjwa katika huo utafiti uliofanyika hawakuchunguzwa magonjwa yasababishwayo na bakteria kutoka kwa wanyama kwenda kwa binadamu?
- Je una maswali yoyote kuhusiana na hayo matokeo ya huo utafiti?

Asante sana kwa ushiriki wako katika utafiti wetu.
